# Targeting Genomic Alterations in Squamous Cell Lung Cancer

**DOI:** 10.3389/fonc.2013.00195

**Published:** 2013-08-05

**Authors:** Kalyan Mantripragada, Humera Khurshid

**Affiliations:** ^1^The Brown University Oncology Research Group, Providence, RI, USA

**Keywords:** EML4-ALK, DDR2, PIK3CA, AKT1, EphA2, LKB1, FGFR1, SOX2

## Abstract

Squamous cell lung cancer causes approximately 400,000 deaths worldwide per year. Identification of specific molecular alterations, such as activating mutations in the epidermal growth factor receptor kinase and echinoderm microtubule-associated protein-like 4/anaplastic lymphoma kinase fusions have led to significant therapeutic gains in patients with adenocarcinoma. However, meaningful therapeutic gains based on the molecular pathobiology of squamous cell lung cancer have not yet been realized. A comprehensive genomic characterization of 178 cases of squamous cell lung cancer has recently been reported. Squamous cell lung cancer appears to be characterized by a broader and more complex group of genomic alterations than adenocarcinoma. In this review, potentially targetable genes or pathways in squamous cell lung cancer are emphasized in relation to available therapeutic agents in development or active clinical trials. This organization of data will provide a framework for development for clinical investigation. Squamous cell lung cancer appears to be characterized by not only driver mutations in candidate genes but also gene copy number alterations resulting in tumor proliferation and survival. Better understanding of these genetic alterations and their use as therapeutic targets will require broad collaboration between industry, government, the cooperative groups, and academic institutions with the ultimate goal of rapid translation of scientific advancement to patient benefit.

## Introduction

Lung cancer is the leading cause of cancer related deaths worldwide. Adenocarcinoma is the most common form of non-small cell lung cancer (NSCLC); the second most common is squamous cell (1, 2). Targeted kinase inhibitors have successfully changed the treatment paradigm for adenocarcinoma. The discovery of epidermal growth factor receptor kinase (EGFR) activating mutations and echinoderm microtubule-associated protein-like 4/anaplastic lymphoma kinase (EML4/ALK) gene translocations in adenocarcinoma has shifted the focus from histological subtypes to targetable mutations (3–6). The EGFR tyrosine kinase inhibitors (TKIs) erlotinib (Tarceva, Basel, Switzerland) (7, 8) and gefitinib (Iressa, AstraZeneca, London, UK) (9, 10) and the ALK inhibitor crizotinib (Xalcori, Pfizer, NY, USA) (4) have significantly improved response rates and progression free survival in patients with lung adenocarcinoma. Gene rearrangement in adenocarcinoma with ROS1, RET also appear to be important clinical targets (11, 12).

In contrast, activating mutations in EGFR and ALK fusions are generally not present in squamous cell lung cancer and targeted agents are usually not effective (13). There are no United States Food and Drug Administration approved targeted therapies for squamous cell lung cancer.

This review describes the genomic alterations associated with squamous cell lung cancer. Emphasis is derived from information recently published from the genomic characterization of squamous cell lung cancers from a cohort of patients as part of The Cancer Genome Atlas (TCGA) project (14). This analysis consisted of samples from 178 patients with previously untreated stage I to stage IV squamous cell lung cancer. Tumor types were characterized by complex genomic alterations, with a mean of 360 exonic mutations, 165 genomic rearrangements, and 323 segments of copy number alteration *per each tumor*. Researchers observed that 96% (171 out of 176) of tumors had one or more mutation in tyrosine kinases, serine/threonine kinases, phosphatidylinositol-3-kinase, and multiple significantly altered pathways. The goal of this review is to provide a framework to apply these advances in the molecular understanding of squamous cell lung cancer to clinical applications. Genomic alterations that are potential targets, and the corresponding therapeutic agent in clinical trials or in development are reviewed.

## Materials and Methods

A literature search was conducted for articles on PubMed published in English language from 1980 to 2012. We used the search term “squamous cell cancer of the lung” with “gene copy number alterations,” “somatic mutations,” “gene amplifications,” and “clinical trials.” We also performed specific searches for the candidate genes reported in genomic studies of squamous cell cancer of the lung with emphasis on drugs currently in clinical trials. For the purpose of this review, targetable genomic alterations are being separated as “mutations” and “amplifications/overexpression” depending on the predominant targetable alteration known with the individual genes.

## Targetable Genomic Alterations

### Mutations

#### DDR2

**Figure 1:**
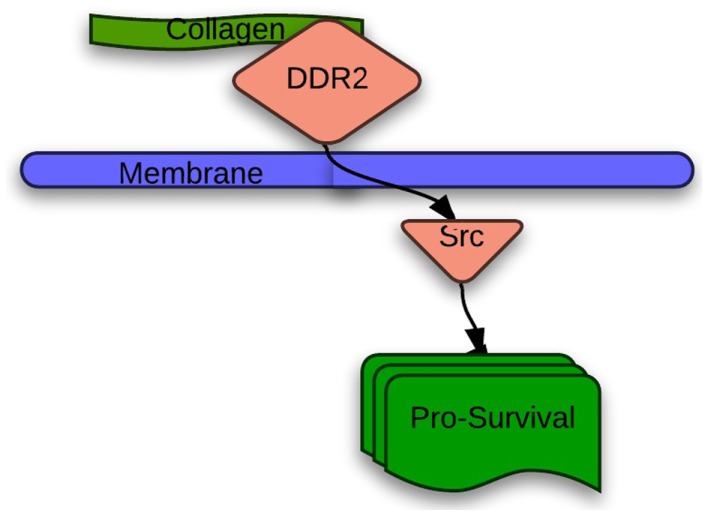
**Proposed DDR2 signaling in lung cancer**.

Discoidin death receptor 2 (DDR2) is a receptor tyrosine kinase activated by binding of its discoidin domain to fibrillar collagen (Figure 1) (15). Apart from its important role in postnatal development and tissue repair, DDR2 also regulates primary and metastatic cancer progression. Though unclear, several downstream signaling pathways have been implicated in DDR2 signaling, including Shp-2, Src, STAT, and MAPK pathways (16). The S768R mutation results in substitution of serine with arginine at position 768 in DDR2. In addition to lung squamous cell cancers, DDR2 mutations are found in cancers of the kidney, breast, endometrium, colon, and brain (17). Hammerman et al. identified oncogenic mutations in the DDR2 kinase gene in 3.8% of the 222 Sanger sequenced lung squamous cell samples (18).

Dasatinib (Sprycel, Bristol-Myers Squibb, NY, USA) is a marketed multitargeted kinase inhibitor that blocks DDR2 (18, 19). Hammerman et al. reported selective *in vitro* killing of squamous cancer cell lines harboring DDR2 mutations by either knockdown of DDR2 with RNA interference or by treatment with dasatinib (18). *In vivo* sensitivity of DDR2 to dasatinib was demonstrated by inhibition of tumors induced into athymic nude mice. As demonstrated in other studies (19) potent inhibitory effect of imatinib on DDR2 induced oncogenic transformation was also reported in this study. However, such inhibition was most shown to be most potent with dasatinib, given concurrent Src inhibition. Hammerman et al. also independently verified the presence of S768R mutation in the pre-treatment tumor sample of a female patient with squamous cell cancer with no EGFR mutation who responded to a combination of dasatinib and erlotinib in a different study (20). Currently this combination is being studied in phase I trials recruiting patients with advanced cancer. Dasatinib monotherapy is being studied in advanced squamous cell lung cancers especially in individuals whose tumors harbor mutations in the DDR2 gene (Table 1). Dasatinib can cause pleural effusions. The Brown University Oncology Group evaluated dasatinib in a phase I study with chemoradiation in an unselected cohort of patients with stage III NSCLC. This trial needed to be terminated due to pulmonary toxicity. Therefore caution is needed when dasatinib is utilized for patients with lung cancer and prior thoracic radiation (21).

**Table 1:** **Targetable genes and ongoing clinical trials in squamous cell carcinoma of the lung**.

Gene	Cytogenetic location	Mechanism of carcinogenesis	Frequency in squamous cell cancer	Frequency in adenocarcinoma	Novel agents	Clinical trial number	Study phase
FGFR1	8p11–12	Amplification	21% (57)	3.4% (57)	NVP-BGJ398	NCT01004224	I
					AZD4547	NCT00979134	I
					E-3810	NCT01283945	I
					ASA404 + carboplatin/paclitaxel/cetuximab	NCT01031212	I
DDR2	1q23.3	Mutation	3.8% (18)	Not reported	Dasatinib + erlotinib	NCT00895128	I
					Dasatinib	NCT01491633	II
						NCT01514864	II
PIK3CA	3q26.3	Mutation	9% (23)	1.5% (24)	BKM120	NCT01068483	I
		Amplification	42–43% (27, 28)	6% (22)	BKM120 + docetaxel	NCT01540253	I
					BKM120 + gefitinib	NCT01570296	I
					BKM120 + carboplatin + paclitaxel	NCT01297452	I
					BKM120 + MEK162	NCT01363232	I
					BKM120 + everolimus	NCT01470209	I
					BYL719	NCT01387321	I
					AZD5363	NCT01226316 NCT01353781	I
					SAR256212 + SAR245409	NCT01436565	I
					SAR256212 + erlotinib	NCT00994123	I/II
					SAR256212 + cabazitaxel/carboplatin/pemetrexed	NCT01447225	I
PTEN	10q23.3	Mutation	10.2% (35)	1.7% (35)	GSK2636771	NCT01458067	I/IIa
AKT1	14q32.32	Mutation	7.1% (45)	0% (45, 46)	GDC-0068 + paclitaxel/docetaxel	NCT01362374	I
					MK-2206 + gefitinib	NCT01147211	I
MET	7p11.4–7qter	Increased expression	52.6% (81)	81.3% (81)	MetMAb + erlotinib	NCT00854308	I
		Mutation	Infrequent	1.5% (86)		NCT01456325	III
					METMAb + paclitaxel + platinum	NCT01519804	II
					PF-02341066 + PF-00299804	NCT01441128	I
					PF-02341066	NCT00585195	I
					EMD1214063	NCT01014936	I
					ARQ197 + pazopanib	NCT01468922	I
					INC280	NCT01324479	I
					SAR125844	NCT01391533	I
					GSK1363089 + erlotinib	NCT01068587	I/II
					X-396	NCT01625234	I
SOX2	3q26.33	Amplification	20% (67)		None		
		Increased expression	90% (68)	21% (68)	None		
EphA2	1p36	Mutation	7% (50)	0% (50)	None		
LKB1	19p13.3	Mutation	19% (55)	34% (55)	None		

### PI3K/PTEN/AKT/mTOR pathway

#### PIK3CA

**Figure 2:**
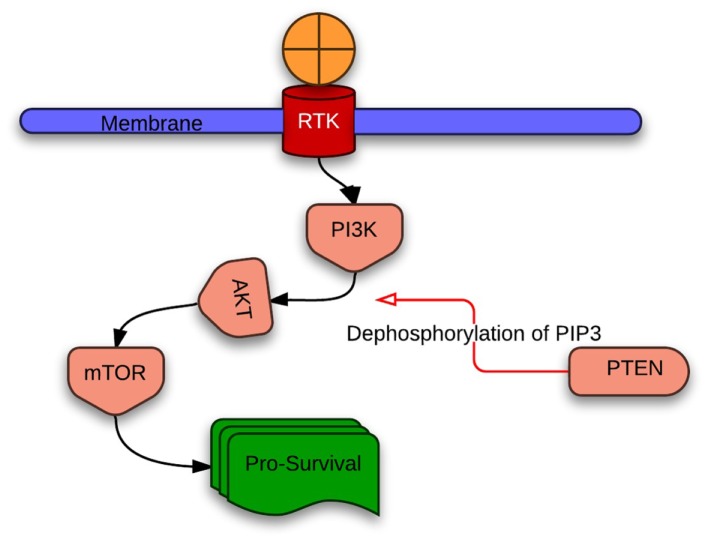
**Simplified sequence of PI3K/PTEN/AKT interaction affecting survival after growth factor binding to receptor tyrosine kinase**. Red arrow indicates inhibitory action.

Phosphatidylinositol-3 kinases (PI3K) belong to a family of heterodimeric lipid kinases. Normally, PI3K converts phosphatidy linositol-3,4-bisphosphate [PI(4,5)P2] to phosphatidylinositol-3,4,5-trisphosphate [PI(3,4,5)P3], which in turn activates downstream AKT/mTOR pathway to regulate growth, survival, and motility. Three classes of PI3K exist; class IA is the only protein found to have somatic mutations in human cancers (22). PI3K contains catalytic and regulatory subunits encoded by separate genes. Mutations mostly occur in the helical and kinase domains of PIK3CA, the gene that encodes the p110α catalytic subunit of PI3K. These mutations are reported in up to 9% of squamous cell lung cancers and are more common in squamous cell than in adenocarcinomas (22–24). Evidence for the oncogenic potential of PIK3CA mutations stems from the association of PI3K with various oncogenes like phosphatase and TENsin homolog (PTEN) and AKT (Figure 2) (25, 26). Amplification of PIK3CA in the 3q locus (3q26) is also a mechanism of oncogene activation in squamous cell lung cancer. Copy number gains of up to 43% in lung squamous cell cancer were demonstrated suggesting that amplifications of PIK3CA are more frequent than mutations (27, 28).

PIK3CA mutations were found to coexist with EGFR mutation in studies by Sun et al. (four of four tumors) and Kawano et al. (three of eight tumors) (24, 29). Activation of PI3K/AKT pathway promotes resistance to EGFR TKIs in EGFR-mutant NSCLC (30). PIK3CA mutations have been detected in about 5% of EGFR-mutant lung cancers with acquired resistance to EGFR-TKI therapy (31).

In a phase I dose-escalation study in advanced solid tumors using BKM120 (Novartis, Basel, Switzerland), an oral pan-Class I PI3K inhibitor, two lung cancer patients were included, and one responded with stable disease (32). PI-103, a dual PI3Kα and mTOR inhibitor showed clear antitumor activity in two gefitinib-resistant NSCLC cell lines, A549 and H460 (33). BYL719 (Novartis, Basel, Switzerland), BKM120 (Novartis, Basel, Switzerland), AZD5363 (AstraZeneca, London, UK), SAR256212 and SAR245409 (Sanofi, Paris, France) are other targeted PIK3CA inhibitors being studied in phase I trials for advanced solid tumors with an alteration of the PIK3CA gene (Table 1). Caution is needed when biologic agents that effect overlapping pathways are utilized together. For example in a Brown University Oncology Group phase I study combining the mTOR inhibitor ridaforolimus with anti-EGFR antibody, severe mucositis developed at ridaforolimus doses typically not associated with this toxicity (NCT01212627).

#### PTEN

Phosphatase and TENsin homolog encodes a lipid-protein phosphatase. By dephosphorylation of PIP3, PTEN negatively regulates PI3K/AKT leading to tumor suppression (34). The R233 mutation in exon 7 results in introduction of a premature stop codon into the PTEN gene resulting in inactivation of PTEN (35). PTEN inactivation therefore increases activity of the PI3K-AKT-mTOR pathway which promotes survival. Incidence of PTEN mutation is about 4–8% in NSCLC (35, 36). Jin et al. analyzed 176 surgically resected NSCLCs. PTEN mutations were only found in smokers and were significantly more frequent in squamous cell lung cancers than in adenocarcinomas (10.2 vs. 1.7%) (35). Similar pattern was observed in the analysis of 173 NSCLC tumors by Lee et al. (36) PTEN inactivation decreases sensitivity of EGFR-mutant lung tumors to EGFR through partial uncoupling of mutant EGFR from downstream signaling leading to EGFR activation and through activation of AKT (37). PTEN loss on the contrary increases sensitivity to PI3K-AKT and FKBP12-rapamycin associated protein (mTOR) inhibitors via increased S6 kinase activity and phosphorylation of ribosomal S6 protein (38) In addition to several studies involving unselected patient population treated with PI3K/AKT inhibitors and mTOR inhibitors, a selected population with PTEN deficiency is being studied with the experimental drug GSK2636771 (GlaxoSmithKline, London, UK; Table 1).

#### AKT1

v-AKT murine thymoma viral oncogene homolog 1 (AKT1 oncogene) encodes a class of serine-threonine protein kinases that are involved in growth, proliferation, angiogenesis, and survival. AKT1 is a downstream regulator of PI3K pathway and is activated by either loss of function of PTEN gene or mutations/amplifications of PIK3CA gene. AKT1 activation requires both plasma membrane translocation and phosphorylation at Thr308 and Ser473 (atleast at Thr308) (39–41) and involves binding of its pleckstrin homology domain (PHD) to PI(3,4,5)P3 followed by Thr308/Set473 phosphorylation or phosphorylation of Thr308 by 3-phosphoinositide-dependent protein kinase-1 (PDK1) (42, 43). By analysis of clinical tumor specimens, Carpten et al. described the E17K mutation (point mutation at nucleotide 49 that results in a lysine substitution for glutamic acid at amino acid 17) in the PHD of AKT1 which constitutively activates the kinase (44). AKT1 activation is oncogenic as evidenced by transformation of cells in culture and induction of leukemia in mice (44). E17K mutations occur in NSCLC at a rate of 1–2% and were identified in 1 of 14 lung squamous cell samples analyzed by Do et al. (45) and 2 of 36 lung squamous cell samples analyzed by Malanga et al. (46).

An open-label, multicenter, Phase Ib dose-escalation combination study using a 3 + 3 design of GDC-0068 (Genentech Inc., South San Francisco, CA, USA), a novel oral selective ATP-competitive AKT inhibitor targeting P13k-Akt-mTOR pathway in metastatic cancer refractory to standard therapy enrolled 23 patients in the GDC-0068 + docetaxel (Taxotere, Sanofi-Aventis, Paris, France) arm and is ongoing. One patient with NSCLC in this arm experienced partial response as evidenced by 32% tumor reduction by modified Response Evaluation Criteria in Solid Tumors criteria (47). Another ongoing phase I study combining the AKT inhibitor MK-2206 (Merck, NJ, USA) with gefitinib in NSCLC patient population enriched for EGFR mutations is recruiting participants (Table 1). The PHD of PDK1 also provides another potential target for drug development.

#### EphA2

The erythropoietin-producing hepatocellular (Eph) family of receptor tyrosine kinases regulates a multitude of physiological and pathological processes. Eph receptors are tyrosine kinases involved in embryonic development (48). Eph A2 is over expressed in NSCLC (49). Mutations in EphA2 although over all rare, are more common in lung squamous cell cancer histology. Both over expression and mutation increase the risk of invasive disease. The mutation G391R in EphA2 confers susceptibility to treatment with rapamycin (Rapamune, Pfizer, NY, USA), an mTOR inhibitor and was seen in 7% of squamous cell lung cancers vs. 0% of other histologies in one report (50). Overexpression of EphA2 also leads to increased phosphorylation of Src.

Dasatinib a multitargeted TKI has activity against EphA2 receptors (51, 52). Therefore EphA2 is a potential target for specific drug development and clinical trials are indicated.

#### LKB1

Liver Kinase B1 (LKB1, also called STK11) is frequently mutated in NSCLC and is thought to act as a tumor-suppressor gene. LKB1 acts as a tumor suppressor by activating AMPK (5′ AMP-activated protein kinase). Loss of LKB1 by point mutation or deletion suppresses AMPK, leading to increased mTOR signaling. LKB1 is thought to function in early tumorigenesis, subsequent differentiation, and the development of metastases (53). Low levels of LKB1 protein were associated with high grades of dysplasia in atypical adenomatous hyperplasia lesions, suggesting that LKB1 has an early role in the development of premalignant lesions of the lung (54). LKB1 mutations (including point mutations and deletions) were found in 34% of adenocarcinomas and 19% of squamous cell lung cancers from 144 human specimens of NSCLC (55). Mutations are more common in Whites as compared to Asians. LKB1 inactivation and KRAS mutation enhanced tumor growth and metastatic potential in mouse models.

Recently researchers at The University of Texas MD Anderson Cancer Center, investigated the effects of LKB1 mutation and mTOR inhibition on cell signaling pathways, measuring protein expression in NSCLC cell lines by reverse phase protein array. LKB1 mutant cell lines have increased insulin-like growth factor receptor (IGFR) activity with higher baseline IGFR1, insulin-like growth factor-binding protein 2 (IGFBP2), and nuclear receptor coactivator 3 (AIB1), suggesting that IGFR may be a potential therapeutic target in LKB1 mutant tumors. In addition, inhibition of the mTOR pathway was shown to upregulate the IGFR pathway, possibly as a feedback mechanism. These results support the investigation of IGFR inhibitors in combination with drugs targeting the mTOR pathway, particularly for tumors bearing LKB1 mutations (56). Currently there are no trials looking at this combination.

## Amplifications/Overexpression

### FGFR1

**Figure 3:**
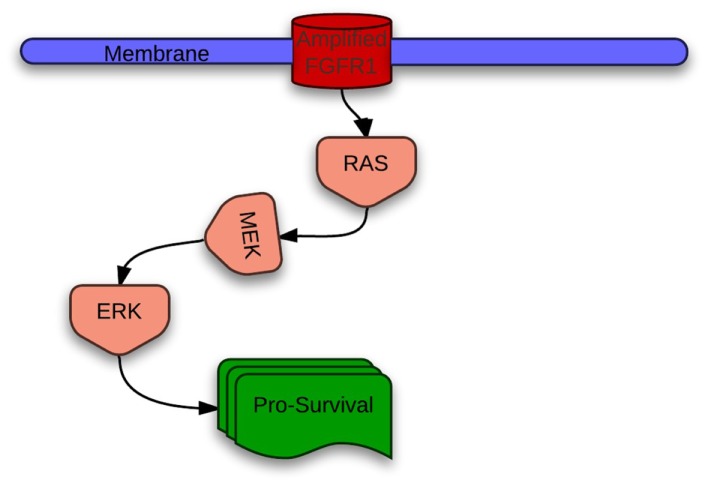
**Predominant intracellular signaling from amplified FGFR1 in lung cancer**.

The fibroblast growth factor receptor type 1 gene (FGFR1) encodes a cell surface tyrosine kinase receptor of the FGFR tyrosine kinase family, which includes four kinases, FGFR1–4. They play a critical role in cell proliferation and survival (Figure 3). Based on single nucleotide polymorphism (SNP) array copy number analysis of 732 samples, Dutt et al. report that the region of chromosome segment 8p11–12 containing FGFR1 is somatically amplified in 21% of lung squamous cell cancers as compared to 3.4% of lung adenocarcinomas (57). This high frequency of FGFR1 amplification in squamous cell lung cancer was confirmed by Weiss et al. (22% in their analysis) using fluorescent *in situ* hybridization (FISH) (58). Lymph node metastases derived from FGFR1-amplified squamous cell cancer also are known to exhibit FGFR1 amplification (59).

Dutt et al. studied the effects of pan-FGFR inhibitor PD173074 (Pfizer, Groton, CT, USA) on NSCLC cell lines. FGFR1-amplified NCI-H1581 cells were sensitive to treatment with PD173074 as assayed by colony formation in soft agar with half maximal inhibitory concentration (IC_50_) in the range of 10–20 nM. In contrast, NCI-H2170 cells with wild type FGFR1 copy number were insensitive to PD173074 (57). Growth dependence of these cell lines on FGFR1 amplification identifies this genetic variation as a high-frequency therapeutic target in squamous cell lung cancer (57, 58, 60).

Genomic and cell line sensitivity studies on cancer cell lines also demonstrated sensitivity of FGFR gene alterations for the pan-FGFR small molecule inhibitor NVP-BGJ398 (Novartis, Basel, Switzerland) (61). In a phase I dose-escalation study in genetically preselected advanced solid tumors, patients received NVP-BGJ398 daily in a 28-day cycle in escalating dose cohorts starting from 5 mg once daily. After cohort 3, patients had to have FGFR1 or FGFR2 amplification or FGFR3 mutation. 26 patients, including three patients with FGFR1-amplified squamous cell cancer were treated. One lung cancer patient with an FGFR1/CEP8 ratio of 2.6 by FISH responded substantially to 100 mg of NVP-BGJ398 as assessed by computed tomography and positron emission tomography (62). NVP-BGJ398 is being studied in another multicenter phase I study. The safety and tolerability of AZD4547 (AstraZeneca, London, UK) in FGFR1 and/or FGFR2 gene amplified solid tumors and FGFR1 gene amplified squamous cell cancer is being studied in an ongoing study. E-3810 (EOS SpA, Milano, Italy), a novel dual-targeted small molecule inhibitor of VEGFR1, 2, 3 and FGFR1 showing strong anti-angiogenic and antitumor activity in preclinical models is currently being studied in a phase I trial in advanced solid tumors (Table 1).

### SOX2 amplification and over expression

SRY (sex determining region)-box 2 (SOX2) protein is an evolutionarily conserved 317 aminoacid transcription factor containing a high mobility group (HMG) box. It is a critical transcription regulator of normal embryonic and neural stem cell function. SOX2 is required for foregut morphogenesis, playing an important role in the normal development of lung and esophagus (63, 64). When somatic DNA alterations develop in these cell lineages, SOX2 is activated, justifying its classification as a lineage survival oncogene (65, 66). SOX2 is expressed in peak genomic amplifications of chromosome segment 3q26.33 that occur in about 20% of squamous cell lung cancers and such expression is independent of the degree of histological differentiation (67). Sholl et al. examined 121 lung tumor specimens for Sox2 protein expression by IHC and demonstrated that SOX2 is strongly and diffusely expressed (extensive SOX2 staining described as immunoreactivity in>50% of tumor cells) in approximately 90% of squamous cell cancers, 21% of adenocarcinomas, and 72% of high grade neuroendocrine tumors (68). Oncogenic mechanisms induced through SOX2 overexpression include promotion of cell migration, anchorage-independent growth, and squamous cell viability by protection from apoptosis (67). Lu et al. have demonstrated that SOX2 overexpression is oncogenic *in vivo*. About 50% mice with upregulated SOX2 developed lung cancer (69). Targeting of SOX2 has not yet entered clinical investigations.

### MET

MNNG-HOS transforming gene (MET-oncogene) was initially cloned from chemically transformed human osteosarcoma-derived cell line and was mapped to chromosome 7 (7p11.4–7qter) (70). The MET-oncogene encodes the tyrosine kinase MET, which is the receptor for hepatocyte growth factor (HGF) or scatter factor (SF). Ligand binding or mutations/gene amplifications in the MET-oncogene, activate MET. Activated MET phosphorylates its substrates, activating multiple downstream pathways involving P13K, RAS-MAPK, and STAT3 (71, 72).

Hepatocyte growth factor-MET signaling mediates biological processes like proliferation, motility angiogenesis, and invasiveness (72–74). MET tyrosine kinases are crucially involved in the invasive growth program and aberrant activation contributes to tumor growth, progression, and metastasis (72, 75, 76). Cells which over-express either HGF or MET are tumorigenic and highly metastatic when implanted into nude mice (77). When MET is switched off, tumors were shown to become less aggressive (78). Germline and somatic mutations in MET-oncogene leading to MET activation were identified in papillary renal cell carcinomas (79). Somatic mutations in NSCLC were first identified by Kong-Beltran et al. (80) who also noted that tumors harboring these mutations were wild type for KRAS, B-raf, EGFR, and HER2. Strong MET expression was observed by immunohistochemistry in 52.6% of squamous cell lung cancer specimens (81). MET amplification is rare in lung cancer but is seen in up to 20% of tumors treated with EGFR TKIs and contributes to EGFR-TKI resistance (82, 83). Due to this reason, several researches have urged that the development of anti-MET therapeutic strategies should be focused on patients with acquired EGFR-TKI resistance (82). High MET expression indicates poor prognosis and may portend development of brain metastases in lung cancer (83).

Inhibitors of ALK and ROS also inhibit MET. A case report of an NSCLC patient with *de novo* MET amplification but no ALK rearrangement who achieved a rapid and durable response to crizotinib indicates that the drug is a potent MET inhibitor (84). A randomized phase II study evaluating anti-MET monoclonal antibody (MetMAb) or placebo in combination with erlotinib in 128 patients with advanced NSCLC has been reported. In the metastatic NSCLC group, the combination of MetMAb and erlotinib resulted in a statistically and clinically significant improvement in both the progression free and overall survivals resulting in a near threefold reduction in the risk of death (85). Several studies are underway evaluating role of ALK inhibitors like X-396 (Xcovery Holding Company, West Palm Beach, FL, USA), MET/HGF TKIs like PF-02341066 (Pfizer, Groton, CT, USA), and onartuzumab/MetMAb (Genentech Inc., South San Francisco, CA, USA) in NSCLC (Table 1).

## Perspective and Future Directions

Lung carcinogenesis is due to the accumulation of mutations in oncogenes, tumor-suppressor genes, and genetic instability genes as well as alteration in gene copy number. Carcinogens such as cigarette smoke are an important cause of mutations in lung cancer especially squamous cell cancer. Squamous cell cancer of the lung is known for its genomic complexity and is characterized by an overall high mutation rate of 8.1 mutations per megabase as evident by the recent comprehensive genomic characterization of squamous cell cancers of the lung by “TCGA Research Network.”

Both driver mutations and pathways affected by gene copy number alterations can be potentially targeted for therapeutic benefit. A driver mutation results in survival advantage and tumor proliferation. It has been estimated that approximately 60% of lung adenocarcinomas will have driver mutations. True driver mutations that are currently targetable appear to be present in a minority of cases of squamous cell lung cancer and include DDR2 (3.8%), BRAF (4%), and EGFR (2%). Thus a single targeted therapy is unlikely to be effective in a large proportion of squamous cell lung cancer patients. However we know that mutations in PIK3CA, AKT, PTEN, EphA2, and LKB1 occur in squamous cell cancer of the lung and although we may not have a specific inhibitor at this point for all, combination of targeted agents or strategies to target downstream intracellular pathways such as the PI3K/PTEN/AKT/mTOR pathway may end up being more effective. Patients with squamous cell cancer of the lung harboring genetic defects that are targetable should be enrolled in clinical trials specific for the target.

The Lung Cancer Mutation Consortium (LCMC) represents the largest national initiative to prospectively examine lung adenocarcinomas with the potential to match patients to the best possible therapies. Targeting uncommon mutations in squamous cell lung cancer, with monotherapy and innovative combinations, will require similar broad collaborations between industry, academia, and government institutions. It is hoped that the dramatic gain in knowledge and understanding of genomic alterations in squamous cell lung cancer will steadily translate into meaningful patient benefit.

## Conflict of Interest Statement

The authors declare that the research was conducted in the absence of any commercial or financial relationships that could be construed as a potential conflict of interest.

## References

[1] Siegel R Naishadham D Jemal A Cancer statistics. CA Cancer J Clin (2013) 63:11–30 10.3322/caac.21166 23335087

[2] Morgensztern D Waqar S Subramanian J Gao F Govindan R Improving survival for stage IV non-small cell lung cancer: a surveillance, epidemiology, and end results survey from 1990 to 2005. J Thorac Oncol (2009) 4:1524–9 10.1097/JTO.0b013e3181ba3634 19752759

[3] Kwak EL Bang YJ Camidge DR Shaw AT Solomon B Maki RG Anaplastic lymphoma kinase inhibition in non-small-cell lung cancer. N Engl J Med (2010) 363:1693–703 10.1056/NEJMoa1006448 20979469 PMC3014291

[4] Shaw AT Yeap BY Solomon BJ Riely GJ Gainor J Engelman JA Effect of crizotinib on overall survival in patients with advanced non-small-cell lung cancer harbouring ALK gene rearrangement: a retrospective analysis. Lancet Oncol (2011) 12:1004–12 10.1016/S1470-2045(11)70232-7 21933749 PMC3328296

[5] Pao W Miller V Zakowski M Doherty J Politi K Sarkaria I EGF receptor gene mutations are common in lung cancers from “never smokers” and are associated with sensitivity of tumors to gefitinib and erlotinib. Proc Natl Acad Sci U S A (2004) 101:13306–11 10.1073/pnas.0405220101 15329413 PMC516528

[6] Lynch TJ Bell DW Sordella R Gurubhagavatula S Okimoto RA Brannigan BW Activating mutations in the epidermal growth factor receptor underlying responsiveness of non-small-cell lung cancer to gefitinib. N Engl J Med (2004) 350:2129–39 10.1056/NEJMoa040938 15118073

[7] Zhou C Wu YL Chen G Feng J Liu XQ Wang C Erlotinib versus chemotherapy as first-line treatment for patients with advanced EGFR mutation-positive non-small-cell lung cancer (OPTIMAL, CTONG-0802): a multicentre, open-label, randomised, phase 3 study. Lancet Oncol (2011) 12:735–42 10.1016/S1470-2045(11)70184-X 21783417

[8] Rosell R Carcereny E Gervais R Vergnenegre A Massuti B Felip E Erlotinib versus standard chemotherapy as first-line treatment for European patients with advanced EGFR mutation-positive non-small-cell lung cancer (EURTAC): a multicentre, open-label, randomised phase 3 trial. Lancet Oncol (2012) 13:239–46 22285168 10.1016/S1470-2045(11)70393-X

[9] Mok TS Wu YL Thongprasert S Yang CH Chu DT Saijo N Gefitinib or carboplatin-paclitaxel in pulmonary adenocarcinoma. N Engl J Med (2009) 361:947–57 10.1056/NEJMoa0810699 19692680

[10] Fukuoka M Wu YL Thongprasert S Sunpaweravong P Leong SS Sriuranpong V Biomarker analyses and final overall survival results from a phase III, randomized, open-label, first-line study of gefitinib versus carboplatin/paclitaxel in clinically selected patients with advanced non-small-cell lung cancer in Asia (IPASS). J Clin Oncol (2011) 29:2866–74 10.1200/JCO.2010.33.4235 21670455

[11] Felip E Gridelli C Baas P Rosell R Stahel R Panel Members Metastatic non-small-cell lung cancer: consensus on pathology and molecular tests, first-line, second-line, and third-line therapy: 1st ESMO Consensus Conference in Lung Cancer; Lugano 2010. Ann Oncol (2011) 22:1507–19 10.1093/annonc/mdr150 21536661

[12] Ju YS Lee WC Shin JY Lee S Bleazard T Won JK A transforming KIF5B and RET gene fusion in lung adenocarcinoma revealed from whole-genome and transcriptome sequencing. Genome Res (2012) 22:436–45 10.1101/gr.133645.111 22194472 PMC3290779

[13] Rekhtman N Paik PK Arcila ME Tafe LJ Oxnard GR Moreira AL Clarifying the spectrum of driver oncogene mutations in biomarker-verified squamous carcinoma of lung: lack of EGFR/KRAS and presence of PIK3CA/AKT1 mutations. Clin Cancer Res (2012) 18:1167–76 10.1158/1078-0432.CCR-11-2109 22228640 PMC3487403

[14] Cancer Genome Atlas Research Network Comprehensive genomic characterization of squamous cell lung cancers. Nature (2012) 489:519–25 10.1038/nature11404 22960745 PMC3466113

[15] Ichikawa O Osawa M Nishida N Goshima N Nomura N Shimada I Structural basis of the collagen-binding mode of discoidin domain receptor 2. EMBO J (2007) 26:4168–76 10.1038/sj.emboj.7601833 17703188 PMC2230669

[16] Ruiz PA Jarai G Collagen I induces discoidin domain receptor (DDR) 1 expression through DDR2 and a JAK2-ERK1/2-mediated mechanism in primary human lung fibroblasts. J Biol Chem (2011) 286:12912–23 10.1074/jbc.M110.143693 21335558 PMC3075638

[17] Vogel WF Abdulhussein R Ford CE Sensing extracellular matrix: an update on discoidin domain receptor function. Cell Signal (2006) 18:1108–16 10.1016/j.cellsig.2006.02.012 16626936

[18] Hammerman PS Sos ML Ramos AH Xu C Dutt A Zhou W Mutations in the DDR2 kinase gene identify a novel therapeutic target in squamous cell lung cancer. Cancer Discov (2011) 1:78–89 10.1158/2159-8274.CD-11-0005 22328973 PMC3274752

[19] Day E Waters B Spiegel K Alnadaf T Manley PW Buchdunger E Inhibition of collagen-induced discoidin domain receptor 1 and 2 activation by imatinib, nilotinib and dasatinib. Eur J Pharmacol (2008) 599:44–53 10.1016/j.ejphar.2008.10.014 18938156

[20] Haura EB Tanvetyanon T Chiappori A Williams C Simon G Antonia S Phase I/II study of the Src inhibitor dasatinib in combination with erlotinib in advanced non-small-cell lung cancer. J Clin Oncol (2010) 28:1387–94 10.1200/JCO.2009.25.4029 20142592 PMC3040065

[21] Khurshid H Dipetrillo T Ng T Mantripragada K Birnbaum A Berz D A phase I study of dasatinib with concurrent chemoradiation for stage III non-small cell lung cancer. Front Oncol (2012) 2:56 10.3389/fonc.2012.00056 22666662 PMC3364482

[22] Yamamoto H Shigematsu H Nomura M Lockwood WW Sato M Okumura N PIK3CA mutations and copy number gains in human lung cancers. Cancer Res (2008) 68:6913–21 10.1158/0008-5472.CAN-07-5084 18757405 PMC2874836

[23] Spoerke JM O’Brien C Huw L Koeppen H Fridlyand J Brachmann RK Phosphoinositide 3-kinase (PI3K) pathway alterations are associated with histologic subtypes and are predictive of sensitivity to PI3K inhibitors in lung cancer preclinical models. Clin Cancer Res (2012) 18:6771–83 10.1158/1078-0432.CCR-12-2347 23136191

[24] Kawano O Sasaki H Endo K Suzuki E Haneda H Yukiue H PIK3CA mutation status in Japanese lung cancer patients. Lung Cancer (2006) 54:209–15 10.1016/j.lungcan.2006.07.006 16930767

[25] Li J Yen C Liaw D Podsypanina K Bose S Wang SI PTEN, a putative protein tyrosine phosphatase gene mutated in human brain, breast, and prostate cancer. Science (1997) 275:1943–7 10.1126/science.275.5308.1943 9072974

[26] Knobbe CB Reifenberger G Genetic alterations and aberrant expression of genes related to the phosphatidyl-inositol-3′-kinase/protein kinase B (Akt) signal transduction pathway in glioblastomas. Brain Pathol (2003) 13:507–18 10.1111/j.1750-3639.2003.tb00481.x 14655756 PMC8095764

[27] Okudela K Suzuki M Kageyama S Bunai T Nagura K Igarashi H PIK3CA mutation and amplification in human lung cancer. Pathol Int (2007) 57:664–71 10.1111/j.1440-1827.2007.02155.x 17803655

[28] Ji M Guan H Gao C Shi B Hou P Highly frequent promoter methylation and PIK3CA amplification in non-small cell lung cancer (NSCLC). BMC Cancer (2011) 11:147 10.1186/1471-2407-11-147 21507233 PMC3098185

[29] Sun Y Ren Y Fang Z Li C Fang R Gao B Lung adenocarcinoma from East Asian never-smokers is a disease largely defined by targetable oncogenic mutant kinases. J Clin Oncol (2010) 28:4616–20 10.1200/JCO.2010.29.6038 20855837 PMC2974342

[30] Engelman JA Mukohara T Zejnullahu K Lifshits E Borrás AM Gale CM Allelic dilution obscures detection of a biologically significant resistance mutation in EGFR-amplified lung cancer. J Clin Invest (2006) 116:2695–706 10.1172/JCI28656 16906227 PMC1570180

[31] Sequist LV Waltman BA Dias-Santagata D Digumarthy S Turke AB Fidias P Genotypic and histologic evolution of lung cancers acquiring resistance to EGFR inhibitors. Sci Transl Med (2011) 3:75ra26 10.1126/scitranslmed.3002003 21430269 PMC3132801

[32] Bendell JC Rodon J Burris HA de Jonge M Verweij J Birle D Phase I, dose-escalation study of [1], an oral pan-Class I PI3K inhibitor, in patients with advanced solid tumors. J Clin Oncol (2012) 30:282–90 10.1200/JCO.2011.36.1360 22162589

[33] Zou ZQ Zhang XH Wang F Shen QJ Xu J Zhang LN A novel dual PI3Kalpha/mTOR inhibitor PI-103 with high antitumor activity in non-small cell lung cancer cells. Int J Mol Med (2009) 24:97–101 19513541 10.3892/ijmm_00000212

[34] Davidson L MacCario H Perera NM Yang X Spinelli L Tibarewal P Suppression of cellular proliferation and invasion by the concerted lipid and protein phosphatase activities of PTEN. Oncogene (2010) 29:687–97 10.1038/onc.2009.384 19915616 PMC2816976

[35] Jin G Kim MJ Jeon HS Choi JE Kim DS Lee EB PTEN mutations and relationship to EGFR, ERBB2, KRAS, and TP53 mutations in non-small cell lung cancers. Lung Cancer (2010) 69:279–83 10.1016/j.lungcan.2009.11.012 20018398

[36] Lee SY Kim MJ Jin G Yoo SS Park JY Choi JE Somatic mutations in epidermal growth factor receptor signaling pathway genes in non-small cell lung cancers. J Thorac Oncol (2010) 5:1734–40 10.1097/JTO.0b013e3181f0beca 20881644

[37] Sos ML Koker M Weir BA Heynck S Rabinovsky R Zander T PTEN loss contributes to erlotinib resistance in EGFR-mutant lung cancer by activation of Akt and EGFR. Cancer Res (2009) 69:3256–61 10.1158/0008-5472.CAN-08-4055 19351834 PMC2849653

[38] Neshat MS Mellinghoff IK Tran C Stiles B Thomas G Petersen R Enhanced sensitivity of PTEN-deficient tumors to inhibition of FRAP/mTOR. Proc Natl Acad Sci U S A (2001) 98:10314–9 10.1073/pnas.171076798 11504908 PMC56958

[39] Andjelkovic M Alessi DR Meier R Fernandez A Lamb NJ Frech M Role of translocation in the activation and function of protein kinase B. J Biol Chem (1997) 272:31515–24 10.1074/jbc.272.50.31515 9395488

[40] Bellacosa A Chan TO Ahmed NN Datta K Malstrom S Stokoe D Akt activation by growth factors is a multiple-step process: the role of the PH domain. Oncogene (1998) 17:313–25 10.1038/sj.onc.1201947 9690513

[41] Stokoe D Stephens LR Copeland T Gaffney PR Reese CB Painter GF Dual role of phosphatidylinositol-3,4,5-trisphosphate in the activation of protein kinase B. Science (1997) 277:567–70 10.1126/science.277.5325.567 9228007

[42] Vivanco I Sawyers CL The phosphatidylinositol 3-kinase AKT pathway in human cancer. Nat Rev Cancer (2002) 2:489–501 10.1038/nrc839 12094235

[43] Vanhaesebroeck B Alessi DR The PI3K–PDK1 connection: more than just a road to PKB. Biochem J (2000) 346:561–76 10.1042/0264-6021:3460561 10698680 PMC1220886

[44] Carpten JD Faber AL Horn C Donoho GP Briggs SL Robbins CM A transforming mutation in the pleckstrin homology domain of AKT1 in cancer. Nature (2007) 448:439–44 10.1038/nature05933 17611497

[45] Do H Solomon B Mitchell PL Fox SB Dobrovic A Detection of the transforming AKT1 mutation E17K in non-small cell lung cancer by high resolution melting. BMC Res Notes (2008) 1:14 10.1186/1756-0500-1-14 18611285 PMC2442881

[46] Malanga D Scrima M De Marco C Fabiani F De Rosa N De Gisi S Activating E17K mutation in the gene encoding the protein kinase AKT1 in a subset of squamous cell carcinoma of the lung. Cell Cycle (2008) 7:665–9 10.4161/cc.7.5.5485 18256540

[47] Saura C Jones S Mateo J Hollebecque A Cleary JM Roda Perez D A phase Ib study of the Akt inhibitor GDC-0068 with docetaxel (D) or mFOLFOX-6 (F) in patients (pts) with advanced solid tumors. J Clin Oncol (2012) 30:3021

[48] Pasquale EB Eph receptor signaling casts a wide net on cell behavior. Nat Rev Mol Cell Biol (2005) 6:462–75 10.1038/nrm1690 15928710

[49] Brannan JM Dong W Prudkin L Behrens C Lotan R Bekele BN Expression of the receptor tyrosine kinase EphA2 is increased in smokers and predicts poor survival in non small cell lung cancer. Clin Cancer Res (2009) 15:4423–30 10.1158/1078-0432.CCR-09-0473 19531623

[50] Faoro L Singleton PA Cervantes GM Lennon FE Choong NW Kanteti R EphA2 mutation in lung squamous cell cancer promotes increased cell survival, cell invasion, focal adhesion, and mammalian target of rapamycin activation. J Biol Chem (2010) 285:18575–85 10.1074/jbc.M109.075085 20360610 PMC2881783

[51] Lombardo LJ Lee FY Chen P Norris D Barrish JC Behnia K Discovery of N-(2-chloro-6-methyl-phenyl)-2-(6-(4-(2-hydroxyethyl)-piperazin-1-yl)-2-methylpyrimidin-4-ylamino)thiazole-5-carboxamide (BMS-354825), a dual Src/Abl kinase inhibitor with potent antitumor activity in preclinical assays. J Med Chem (2004) 47:6658–61 10.1021/jm049486a 15615512

[52] Chang Q Jorgensen C Pawson T Hedley DW Effects of dasatinib on EphA2 receptor tyrosine kinase activity and downstream signalling in pancreatic cancer. Br J Cancer (2008) 99:1074–82 10.1038/sj.bjc.6604676 18797457 PMC2567084

[53] Shah U Sharpless NE Hayes DN LKB1 and lung cancer: more than the usual suspects. Cancer Res (2008) 68:3562–5 10.1158/0008-5472.CAN-07-6620 18483235

[54] Ghaffar H Sahin F Sanchez-Cepedes M Su GH Zahurak M Sidransky D LKB1 protein expression in the evolution of glandular neoplasia of the lung. Clin Cancer Res (2003) 9:2998–3003 12912948

[55] Ji H Ramsey MR Hayes DN Fan C McNamara K Kozlowski P LKB1 modulates lung cancer differentiation and metastasis. Nature (2007) 448:807–10 10.1038/nature06030 17676035

[56] Yilmaz E Averett L Diao BL Giri U Gudikote J Fan YH Use of proteomic analysis of LKB1/AMPK/mTOR pathways to identify IGF-1R pathway upregulation with LKB1 loss or mTOR inhibition in NSCLC: Implications for targeted combinations. J Clin Oncol (2012) 30:10612

[57] Dutt A Ramos AH Hammerman PS Mermel C Cho J Sharifnia T Inhibitor-sensitive FGFR1 amplification in human non-small cell lung cancer. PLoS ONE (2011) 6:e20351 10.1371/journal.pone.0020351 21666749 PMC3110189

[58] Weiss J Sos ML Seidel D Peifer M Zander T Heuckmann JM Frequent and focal FGFR1 amplification associates with therapeutically tractable FGFR1 dependency in squamous cell lung cancer. Sci Transl Med (2010) 2:62ra93 10.1126/scitranslmed.3001451 21160078 PMC3990281

[59] Göke F Franzen A Menon R Goltz D Kirsten R Boehm D Rationale for treatment of metastatic squamous cell carcinoma of the lung using fibroblast growth factor receptor inhibitors. Chest (2012) 142:1020–6 10.1378/chest.11-2943 22499828

[60] Turner NC Seckl MJ A therapeutic target for smoking-associated lung cancer. Sci Transl Med (2010) 2:62s56 10.1126/scitranslmed.3001942 21160076

[61] Guagnano V Kauffmann A Wohrle S Stamm C Ito M Barys L FGFR genetic alterations predict for sensitivity to NVP-BGJ398, a selective pan-FGFR inhibitor. Cancer Discov (2012) 2:1118–33 10.1158/2159-8290.CD-12-0210 23002168

[62] Wolf J LoRusso PM Camidge RD Perez JM Tabernero J Hidalgo M A phase I dose escalation study of NVP-BGJ398, a selective pan FGFR inhibitor in genetically preselected advanced solid tumors. Cancer Res (2012) 72:LB122 10.1158/1538-7445.AM2012-LB-122

[63] Que J Luo X Schwartz RJ Hogan BL Multiple roles for Sox2 in the developing and adult mouse trachea. Development (2009) 136:1899–907 10.1242/dev.034629 19403656 PMC2680112

[64] Gontan C de Munck A Vermeij M Grosveld F Tibboel D Rottier R Sox2 is important for two crucial processes in lung development: branching morphogenesis and epithelial cell differentiation. Dev Biol (2008) 317:296–309 10.1016/j.ydbio.2008.02.035 18374910

[65] Garraway L Sellers W Lineage dependency and lineage-survival oncogenes in human cancer. Nat Rev Cancer (2006) 6:593–602 10.1038/nrc1972 16862190

[66] Bass AJ Watanabe H Mermel CH Yu S Perner S Verhaak RG SOX2 is an amplified lineage-survival oncogene in lung and esophageal squamous cell carcinomas. Nat Genet (2009) 41:1238–42 10.1038/ng.465 19801978 PMC2783775

[67] Hussenet T Dali S Exinger J Monga B Jost B Dembele D SOX2 is an oncogene activated by recurrent 3q26.3 amplifications in human lung squamous cell carcinomas. PLoS ONE (2010) 5:e8960 10.1371/journal.pone.0008960 20126410 PMC2813300

[68] Sholl LM Long KB Hornick JL Sox2 expression in pulmonary non-small cell and neuroendocrine carcinomas. Appl Immunohistochem Mol Morphol (2010) 18:55–61 10.1097/PAI.0b013e3181b16b88 19661786

[69] Lu Y Futtner C Rock JR Xu X Whitworth W Hogan BL Evidence that SOX2 overexpression is oncogenic in the lung. PLoS ONE (2010) 5:e11022 10.1371/journal.pone.0011022 20548776 PMC2883553

[70] Cooper CS Park M Blair DG Tainsky MA Huebner K Croce CM Molecular cloning of a new transforming gene from a chemically transformed human cell line. Nature (1984) 311:29–33 10.1038/311029a0 6590967

[71] Furge KA Zhang YW Vande Woude GF Met receptor tyrosine kinase: enhanced signaling through adapter proteins. Oncogene (2000) 19:5582–9 10.1038/sj.onc.1203859 11114738

[72] Birchmeier C Birchmeier W Gherardi E Vande Woude GF Met, metastasis, motility and more. Nat Rev Mol Cell Biol (2003) 4:915–25 10.1038/nrm1261 14685170

[73] Rubin JS Chan AM Bottaro DP Burgess WH Taylor WG Cech AC A broad-spectrum human lung fibroblast-derived mitogen is a variant of hepatocyte growth factor. Proc Natl Acad Sci U S A (1991) 88:415–9 10.1073/pnas.88.2.415 1824873 PMC50821

[74] Bussolino F Di Renzo MF Ziche M Bocchietto E Olivero M Naldini L Hepatocyte growth factor is a potent angiogenic factor which stimulates endothelial cell motility and growth. J Cell Biol (1992) 119:629–41 10.1083/jcb.119.3.629 1383237 PMC2289675

[75] Giordano S Corso S Conrotto P Artigiani S Gilestro G Barberis D The semaphorin 4D receptor controls invasive growth by coupling with Met. Nat Cell Biol (2002) 4:720–4 10.1038/ncb843 12198496

[76] Benvenuti S Comoglio PM The MET receptor tyrosine kinase in invasion and metastasis. J Cell Physiol (2007) 213:316–25 10.1002/jcp.21183 17607709

[77] Rong S Segal S Anver M Resau JH Vande Woude GF Invasiveness and metastasis of NIH 3T3 cells induced by Met-hepatocyte growth factor/scatter factor autocrine stimulation. Proc Natl Acad Sci U S A (1994) 91:4731–5 10.1073/pnas.91.11.4731 8197126 PMC43862

[78] Lutterbach B Zeng Q Davis LJ Hatch H Hang G Kohl NE Lung cancer cell lines harboring MET gene amplification are dependent on Met for growth and survival. Cancer Res (2007) 67:2081–8 10.1158/0008-5472.CAN-06-3495 17332337

[79] Schmidt L Duh FM Chen F Kishida T Glenn G Choyke P Germline and somatic mutations in the tyrosine kinase domain of the MET proto-oncogene in papillary renal carcinomas. Nat Genet (1997) 16:68–73 10.1038/ng0597-68 9140397

[80] Kong-Beltran M Seshagiri S Zha J Zhu W Bhawe K Mendoza N Somatic mutations lead to an oncogenic deletion of met in lung cancer. Cancer Res (2006) 66:283–9 10.1158/0008-5472.CAN-05-2749 16397241

[81] Cai YR Zhang HQ Qu Y Mu J Zhao D Zhou LJ Expression of MET and SOX2 genes in non-small cell lung carcinoma with EGFR mutation. Oncol Rep (2011) 26:877–85 10.3892/or.2011.1349 21687954

[82] Cappuzzo F Janne PA Skokan M Finocchiaro G Rossi E Ligorio C MET increased gene copy number and primary resistance to gefitinib therapy in non-small-cell lung cancer patients. Ann Oncol (2009) 20:298–304 10.1093/annonc/mdn635 18836087 PMC2733067

[83] Benedettini E Sholl LM Peyton M Reilly J Ware C Davis L Met activation in non-small cell lung cancer is associated with de novo resistance to EGFR inhibitors and the development of brain metastasis. Am J Pathol (2010) 177:415–23 10.2353/ajpath.2010.090863 20489150 PMC2893683

[84] Ou SH Kwak EL Siwak-Tapp C Dy J Bergethon K Clark JW Activity of crizotinib (PF02341066), a dual mesenchymal-epithelial transition (MET) and anaplastic lymphoma kinase (ALK) inhibitor, in a non-small cell lung cancer patient with de novo MET amplification. J Thorac Oncol (2011) 6:942–6 10.1097/JTO.0b013e31821528d3 21623265

[85] Spigel DR Ervin TJ Ramlau R Daniel DB Goldschmidt JH Blumenschein GR Final efficacy results from OAM4558g, a randomized phase II study evaluating MetMAb or placebo in combination with erlotinib in advanced NSCLC. J Clin Oncol (2011) 29:7505

[86] Seo JS Ju YS Lee WC Shin JY Lee JK Bleazard T The transcriptional landscape and mutational profile of lung adenocarcinoma. Genome Res (2012) 22:2109–19 10.1101/gr.145144.112 22975805 PMC3483540

